# Genetic and geographic population structure in the malaria vector, *Anopheles farauti*, provides a candidate system for pioneering confinable gene-drive releases

**DOI:** 10.1038/s41437-024-00677-2

**Published:** 2024-03-18

**Authors:** Luke Ambrose, Scott L. Allen, Charlie Iro’ofa, Charles Butafa, Nigel W. Beebe

**Affiliations:** 1 School of the Environment, University of Queensland, St Lucia, Brisbane, QLD Australia; 2 Solomon Islands Ministry of Health, Honiara, Guadalcanal Solomon Islands

**Keywords:** Genetic variation, Phylogenetics

## Abstract

Indoor insecticide applications are the primary tool for reducing malaria transmission in the Solomon Archipelago, a region where *Anopheles farauti* is the only common malaria vector. Due to the evolution of behavioural resistance in some *An*. *farauti* populations, these applications have become less effective. New malaria control interventions are therefore needed in this region, and gene-drives provide a promising new technology. In considering developing a population-specific (local) gene-drive in *An. farauti*, we detail the species’ population genetic structure using microsatellites and whole mitogenomes, finding many spatially confined populations both within and between landmasses. This strong population structure suggests that *An. farauti* would be a useful system for developing a population-specific, confinable gene-drive for field release, where private alleles can be used as *Cas9* targets. Previous work on *Anopheles gambiae* has used the *Cardinal* gene for the development of a global population replacement gene-drive. We therefore also analyse the *Cardinal* gene to assess whether it may be a suitable target to engineer a gene-drive for the modification of local *An. farauti* populations. Despite the extensive population structure observed in *An. farauti* for microsatellites, only one remote island population from Vanuatu contained fixed and private alleles at the *Cardinal* locus. Nonetheless, this study provides an initial framework for further population genomic investigations to discover high-frequency private allele targets in localized *An. farauti* populations. This would enable the development of gene-drive strains for modifying localised populations with minimal chance of escape and may provide a low-risk route to field trial evaluations.

## Introduction

Malaria is a plague on humanity and disproportionally affects those living in poverty, with most morbidity and mortality occurring in Sub-Saharan Africa (World Health Organization 2021a). An estimated 241 million cases of malaria (627,000 deaths) were recorded in 2020, increasing from 227 million reported in 2019. The Southwest Pacific region (New Guinea and the Solomon Archipelago) is second to Africa in terms of malaria morbidity and mortality, as essentially all people live with the risk of malaria infection (World Health Organization 2021a). Despite enormous investment so far this century, the incidence of malaria in this region has been increasing. Ostensibly, since 2015 malaria has increased in Papua New Guinea (PNG) and the Solomon Islands by 50 and 100% respectively (World Health Organization 2021a).

The only vector control interventions currently endorsed widely by the WHO—and highly effective against late night and indoor feeding malaria vector species – are long-lasting insecticidal nets (LLINs) and indoor residual spraying (IRS) (World Health Organization 2022). However, the primary malaria vector in the Southwest Pacific, *Anopheles farauti* (Laveran 1902) has been shown in the Solomon Archipelago to rapidly evolve behavioural resistance to these indoor interventions (Taylor 1975b; Bugoro et al. 2011, 2014; Russell et al. 2016). This adaptation results from a shift in the time of night that the species feeds, changing from biting through the night to primarily feeding earlier outdoors, weakening the efficacy of indoor insecticide-control tools. Despite IRS being highly effective on other vector species in the Solomon Islands like *An. koliensis* and *An. punctulatus* (Taylor, 1975a; Bugoro et al. 2011, 2014; Russell et al. 2016), the behavioural adaptation of *An. farauti* likely contributed to the failure of the Global Malaria eradication campaign (Taylor 1975a) and this early evening feeding behaviour has been reinforced with the rollout of long-lasting insecticide treated nets (LLINs) (Bugoro et al. 2014; Russell et al. 2016). As *An. farauti* is the only malaria vector species in Vanuatu, the primary vector in the Solomon Islands and the most significant vector in coastal New Guinea and on all of its islands (Beebe et al. 2015), new vector control strategies are required to control this outdoor, early evening feeding mosquito.

Although insecticidal mosquito control remains the primary tool for reducing malaria transmission (World Health Organization 2021a), other tools are being developed such as the use of gene-drives as genetic biocontrol technologies (Bier 2022). The concept of genetically modifying mosquitoes to reduce malaria was initiated in 1991 at a meeting sponsored by the MacArthur Foundation and WHO-Tropical Disease Research programme (Ridley and Fletcher 2008). This resulted in a 20-year strategic research plan to develop technologies for genetic modification of the malaria transmitting mosquito, *An. gambiae*, to render it incapable of harbouring or transmitting *Plasmodium* spp. parasites. Although the goals of this plan were not achieved in the anticipated time frame, the recent development of precise genetic modification using CRISPR-*Cas9* (Ran et al. 2013; Jiang and Doudna 2017), has led to the development of gene-drive technologies in the African malaria vector *Anopheles gambiae* which could help to reduce the burden of malaria and other mosquito-borne pathogens (Gantz et al. 2015; Hammond et al. 2016; Kyrou et al. 2018; Nash et al. 2019, 2022; Adolfi et al. 2020; Carballar-Lejarazú et al. 2020; Simoni et al. 2020; Hoermann et al. 2021; Ellis et al. 2022).

In 2021, the WHO-Tropical Disease Research Centre published a guidance framework for developing and using genetically modified mosquitoes (GMMs) as potential tools to reduce the transmission of diseases such as malaria (World Health Organization 2021b). Homing-based *Cas9* gene-drive systems can elicit super-mendelian inheritance of desirable genetic elements in the offspring of individuals carrying a ‘drive allele’ (Bier 2022), and enable the engineering of gene-drive organisms for either population eradication/suppression (Hammond et al. 2016; Kyrou et al. 2018; Simoni et al. 2020) or modification (Nash et al. 2019; Adolfi et al. 2020; Carballar-Lejarazú et al. 2020; Hoermann et al. 2021; Leung et al. 2022). They achieve this by the insertion of a gene encoding a *Cas* enzyme which is expressed in the germline of individuals carrying the drive allele as well as a co-expressed short guide RNA (sgRNA) which ‘instructs’ the *Cas* enzyme the specific genomic location at which to cut (Verkuijl et al. 2022). There are several different Cas enzymes each of which require a specific protospacer adjacent motif (PAM) to be adjacent to the sgRNA target location (Gleditzsch et al. 2019). After cutting takes place at the target genomic location, the drive allele is copied into the alternative allele by one of the cells’ constitutive repair mechanisms, known as homology directed repair (HDR), resulting in cells homozygous for the drive allele (Verkuijl et al. 2022).

Genetic biocontrol strategies such as gene-drives may be viable alternatives to traditional approaches for reducing the burden of malaria in the Southwest Pacific. However, there are major regulatory barriers to taking gene-drive technologies into the field, including their potential to spread across geo-political borders (World Health Organization 2021b; James et al. 2023). One possible way to overcome this problem when initially trialling these new technologies is to design population specific drives that would be confined to geographically restricted populations (for example see Willis and Burt 2021; Sudweeks et al. 2019). To achieve this, the species to be modified must have suitably strong and defined population structure.

Detailed knowledge of the population genetic and geographical structure of *An. farauti* is essential prior to developing strategies for engineering gene-drive technologies in the species. Previous work using single locus Sanger sequencing data on *An. farauti* has shown strong population structure within and between the major landmasses as well as between the smaller islands where it occurs (Ambrose et al. 2012). More recently, a microsatellite and mitochondrial study from the Solomon Archipelago has also revealed strong genetic and geographic structure through these islands with some discordance identified in the location and strength of genetic breaks between mitochondrial and microsatellite markers (Ambrose et al. 2014). In this new study we aim to build on previous work by characterizing the population structure of *An. farauti* throughout most of its range in the Southwest Pacific using nuclear microsatellites. As our previous work identified mitochondrial introgression between species in the *An. farauti* complex (Ambrose et al. 2012), we also generate whole mitogenomes (mitochondrial genomes) for *An. farauti* and its two closest sympatric relatives, *An. irenicus* and *An. hinesorum*, with the aim to further evaluate mitochondrial relationships between these species. Finally, we aim to assess the suitability of the *Cardinal* gene-based pCO-37 gene-drive candidate system (Carballar-Lejarazú et al. 2020), for utility as a potential gene-drive target for confinable and local population modification in *An. farauti*. We hypothesise that there will be spatially confined private alleles at the *Cardinal* locus, present in geographically restricted populations of this highly structured species that may provide options for localised future field trials.

## Materials and methods

### Microsatellite amplification and scoring

**Fig. 1:**
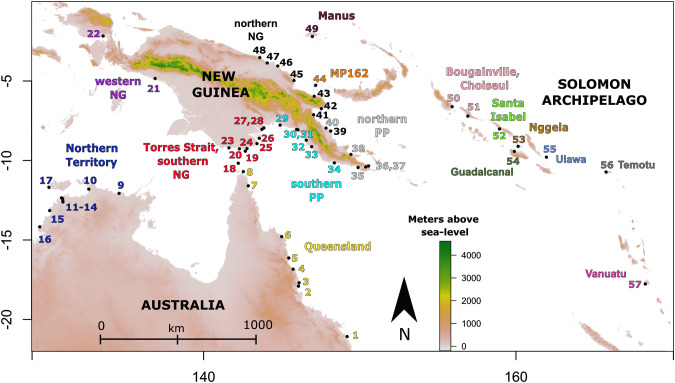
Map of *Anopheles farauti* collection sites. *Anopheles farauti* sites sampled in this study are shown on the above map. Site numbers are consistent with those presented in Table 1 and coloured by genetic groups inferred by STRUCTURE analysis (Fig. 2B). Qld Queensland, NT Northern Territory, TI Torres Strait, sNG southern New Guinea, wNG western New Guinea, sPP southern Papuan Peninsula, nPP northern Papuan Peninsula, nNG northern New Guinea, Manus Manus Island, Bou Bougainville, Choi Choiseul, Isa Santa Isabel, Ngg Nggela, Gua Guadalcanal, Ula Ulawa, Tem Temotu, Van Vanuatu.

Genomic DNA was extracted from field collected samples using the same methods as in Ambrose et al. 2012 and identified as *An. farauti* using a Polymerase Chain Reaction-Restriction Fragment Length Polymorphism (PCR RFLP) of the internal transcribed spacer 2 species diagnostic (Beebe and Saul 1995)—see Table 1 and Fig. 1 for sampling information. Microsatellite primers previously developed and used for the amplification of *An. farauti* from the Solomon Archipelago (Ambrose et al. 2014) were used to amplify 12 microsatellite loci from Australian, New Guinean and Solomon Archipelago populations of *An. farauti*. For sites from Papua province (Indonesia), we were only able to amplify 11 loci, probably due to the poor condition of DNA in samples used (these were long-term dried specimens not preserved in ethanol). Allele size for microsatellites were scored manually using the software GeneMarker (Hulce et al. 2011). Genomic locations of each microsatellite locus were also identified by performing local BLAST searches of primers used against the *An. farauti* reference genome which was downloaded from VectorBase (‘Vectorbase: Bioinformatic resources for invertebrate vectors of human pathogens’ 2021). Any samples missing data for more than four markers were excluded from further analysis.

**Table 1:** Sampling information—*Anopheles farauti*.

Region	Population	Site	Site code	N (MSATS)	N (CARD)^	COORDINATES
AUSTRALIA	Qld	1	(Mac)	–	3^	−21.074, 149.193
		2	(Q47)	–	4	−17.903, 146.07
		3	(Cowley)	8	–	−17.693, 146.111
		4	(CNS)	–	10^	−16.840, 145.730
		5	(NB)	16	–	−16.134, 145.452
		6	(QLD845)	5	–	−14.788, 144.995
		7	(Q885)	2	3, 2^	−11.594, 142.851
		8	(QLD673)	–	2^	−10.7, 142.533
	NT	9	(NT103, NT4)	6	4^	−12.067, 134.567
		10	(NT130)	5	–	−11.804, 132.625
		11	(Cas)	–	2^	−12.354, 130.871
		12	(LD)	5	–	−12.610, 130.964
		13	(PC)	–	1^	−12.492, 130.955
		14	(HJ)	–	3	−12.384, 130.936
		15	(NT2124)	–	1^	−13.15, 130.117
		16	(NT512)	4	–	−14.174, 129.476
		17	(NT1680)	–	2^	−11.683, 130.067
TORRESSTRAIT	TI	18	(TI9, TI324)	12	4,4^	−10.172, 142.210
		19	(Sai)	9	–	−9.417, 142.66
		20	(413)	–	2^	−9.267, 142.283
NEW GUINEA	wNG	21	(Tim)	13	–	−4.843, 136.878
		22	(Bin)	5	–	−2.171, 133.541
	sNG	23	(WP2)	9	–	−9.204, 141.610
		24	(92-8)	14	–	−9.250, 142.769
		25	(92-15)	19	5^	−8.938, 143.409
		26	(92-17)	19	–	−8.601, 143.558
		27	(GR20)	13	–	−8.043, 143.723
		28	(GR21)	15	5^	−7.951, 143.850
	sPP	29	(GR14)	26	10^	−7.777, 144.889
		30	(CP5)	25	5^	−8.049, 145.951
		31	(CP39)	2	3	−8.072, 146.040
		32	(CP70)	5	–	−8.724, 146.569
		33	(CP54)	15	–	−9.131, 146.913
		34	(CP124)	5	–	−10.150, 148.368
	nPP	35	(CP83)	10	–	−10.439, 149.892
		36	(CP100)	10	4	−10.407, 150.381
		37	(CP99)	10	8^	−10.348, 150.565
		38	(CP171)	13	–	−9.620, 149.432
		40	(LR173)	11	–	−7.958, 147.837
	nNG	39	(LR176)	9	6, 6^	−8.148, 148.137
		41	(LR88)	14	2, 2^	−7.123, 147.041
		42	(LR148)	3	–	−6.741, 147.535
		43	(LR9)	4	1^	−5.959, 147.079
		44	(MP162)	9	–	−5.268, 147.178
		45	(MP131)	5	–	−4.968, 145.782
		46	(MP94, MP95)	13	2^	−4.056, 144.741
		47	(SR92)	10	4^	−3.865, 144.065
		48	(SR85)	10	–	−3.531, 143.589
PACIFIC ISLANDS	Manus	49	–	6	4^	−2.205, 146.962
	Bougainville	50	(OPB)	17	5, 10^	−6.613, 155.922
	Choiseul	51	–	6	2	−7.21, 156.931
	Santa Isabel	52	(IPP)	20	–	−8.016, 158.961
	Nggela	53	(TU)	27	–	−9.109, 160.154160.154160.154160.154
	Guadalcanal	54	(JB, GUA)	94	6, 19^	−9.431, 159.911
	Ulawa	55	–	10	5, 5^	−9.791, 161.970
	Temotu	56	(SL)	29	5^	−10.723, 165.793
	Vanuatu	57	(Tana)	12	7, 5^	−17.769, 168.309
TOTAL				613

### Microsatellite population structure and diversity

**Fig. 2:**
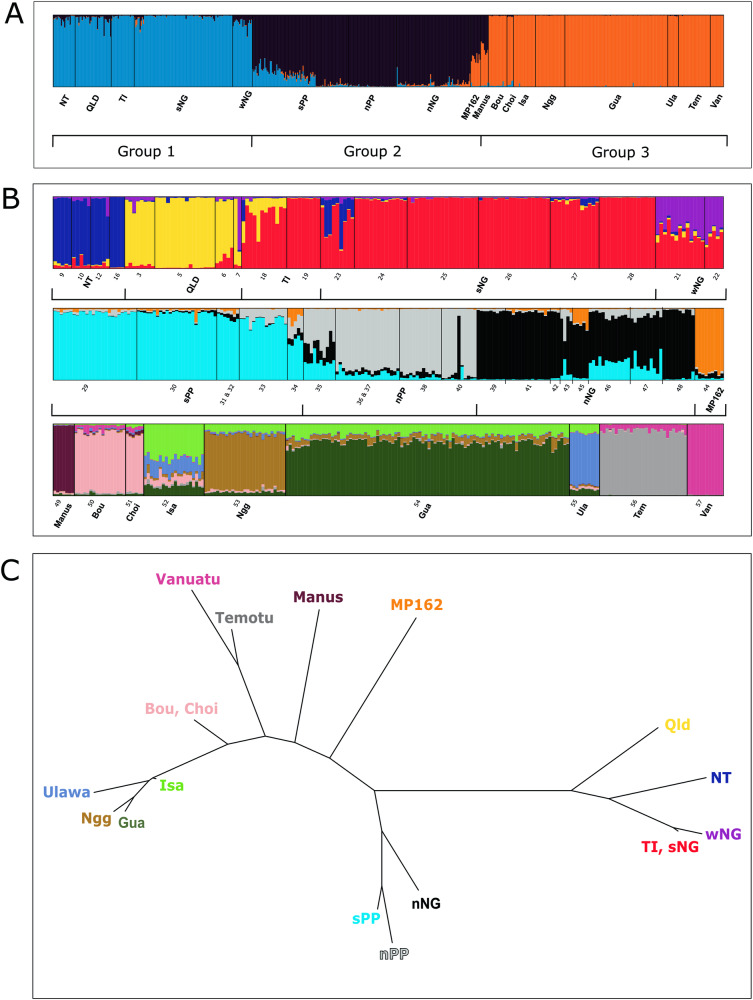
*Anopheles farauti* microsatellite population structure and relationships—Bayesian clustering (STRUCTURE) and Jost’s D. **A** Shows STRUCTURE results for the full dataset (K3). **B** Shows results for STRUCTURE analyses run on the three groups identified by STRUCTURE analysis shown in (**A**). Top panel (**B**) shows results from Group 1 at K4. Middle panel (**B**) shows results from Group 2 at K4. Bottom panel (**B**) shows results from Group 3 at K8. Each vertical bar represents an individual and bar colours show percentage ancestry assigned by the model. **C** Shows a neighbour joining tree generated from pairwise Jost’s D values as a way to visualise relatedness between populations. Population abbreviations and sites are used as in Table 1 and through the manuscript: NT Northern Territory, Australia, Qld Queensland, Australia, TI Torres Strait Islands, sNG southern New Guinea, wNG western New Guinea, sPP southern Papuan Peninsula, nPP northern Papuan Peninsula, nNG northern New Guinea, Manus Manus Island, Bou Bougainville Island, Choi Choiseul Island, Isa Santa Isabel Island, Ngg Nggela Island, Gua Guadalcanal Island, Ulawa Ulawa Island, Tem Temotu, Van Vanuatu.

We assessed population structure using a combination of Bayesian clustering (STRUCTURE (Pritchard et al. 2000)) and multivariate (DAPC (Jombart et al. 2010)) methods as well as fixation indices (Jost’s D (Jost 2008)). Initially, we ran STRUCTURE on all individuals genotyped for 11 microsatellite loci (*n* = 613) to identify broad scale population structure. For this, STRUCTURE was run for 200,000 generations of burn-in and 100,000 generations of sampling, using the admixture model and location priors based on sampling site (10 iterations of K1 to K10). The output from this analysis was the run through the CLUMPAK (Kopelman et al. 2015) server to identify the major mode from the analysis, as well as the optimal K based on both Evanno (Evanno et al. 2005) and Likelihood (Pritchard et al. 2009) methods. Based on the results of this analysis we then separated the data into the three groups identified in the initial STRUCTURE analysis (Fig. 2). The groups identified in the initial STRUCTURE analysis are: (Group 1) Australia, Torres Strait, southern New Guinea and western New Guinea, (Group 2) Papuan peninsula and northern New Guinea, and (Group 3) Manus, Solomon Archipelago, Temotu and Vanuatu. We then ran STRUCTURE on each of these groups separately for 10 iterations from K1 to K10 using the admixture model with location priors (200,000 generation burn-in, 100,000 generation sample). Group 1 was run with 11 loci (*n* = 178), group 2 with 12 loci (*n* = 209) and group 3 with 12 loci (*n* = 222). These results were again run through the CLUMPAK (Kopelman et al. 2015) server to identify the most likely population structure in each of the three groups.

Discriminant Analysis of Principal Components (DAPC) was performed on the microsatellite data in R (R Core Team 2013) using the package adegenet (Jombart 2008). As DAPC requires groups to be defined, we assigned individuals to groups based on the results of STRUCTURE analyses performed on each group separately. We used the “xvalDAPC” function to determine the optimal number of principal components to retain in each DAPC with “training.set” set to 0.9 and “n.rep” to 100. Based on the outcome of this function, we retained 30 PCs for group 1, 40 PCs for group 2 and 20 PCs for group 3. We assessed microsatellite diversity in each population (defined by STRUCTURE) by calculating the number of alleles for each locus, number of private alleles (alleles that are unique to a particular group) and allelic richness both overall and for each marker. An analysis of molecular variance (AMOVA) (Excoffier et al. 1992) was run on the full dataset using the poppr R package (Kamvar et al. 2014) with strata defined at the regional (group), population (based on STRUCTURE results), site and individual levels. Finally, we assessed differentiation between the populations by calculating pairwise Jost’s D values in the R package mmod (Winter 2012) from which we built an unrooted neighbour-joining phylogeny using the R package ape (Paradis and Schliep 2019).

### Whole mitogenome and *Cardinal* sequence analysis

Whole mitogenomes were assembled from 246 individuals and variants were called from four species of the Punctulatus Group (one *Anopheles punctulatus* – outgroup, 77 *Anopheles hinesorum*, 158 *An. farauti* and 10 *An. irenicus*). DNA extractions of these individuals were sequenced on Illumina HiSeq machines following Truseq nano library generation (paired end 100 bp libraries with 350 bp inserts). Sequencing and library preparation were outsourced to Macrogen (Korea) and Novogene (China). Initially following sequencing, we performed quality control using the program fastQC (Andrews 2010), mapping to *Anopheles hinesorum* mito-genome (NCBI reference NC_020769.1 (Logue et al. 2013)) using BWA mem (version 0.7.17-r1188, default settings) (Li 2013), and removed PCR duplicates using MarkDuplicates (version 2.26.6, default settings except REMOVE DUPLICATES was set to True) in picard (GATK) (McKenna et al. 2010) Following duplicate removal, we genotyped individuals using the mpileup (baseQual = 20, mapQual=20) and call (multiallelic-caller) functions in bcftools (Danecek et al. 2021). Finally, we generated fasta files from the vcfs generated using the ‘consensus’ function in bcftools (default settings) and aligned sequences using Clustal W (Thompson et al. 1994) in Geneious Prime (Kearse et al. 2012). A consensus maximum likelihood phylogeny was built using the IQ tree webserver (Nguyen et al. 2015) which used ModelFinder (Kalyaanamoorthy et al. 2017) to identify the best-fit nucleotide substitution model (TN + F + R8) and ultrafast bootstrapping (Hoang et al. 2018) was performed for 1000 bootstrap replicates.

We identified the location of the *Cardinal* gene in the *An. farauti* reference genome by performing a local BLAST of the homology arms from the pCO-37 plasmid against the *An. farauti* reference genome. We then isolated homologous sequences from the *An. farauti* reference genome and from genomes of closely related species previously sequenced (Ambrose et al. 2022). These sequences were aligned using Clustal W and used to design primers with the Primer3 (Rozen and Skaletsky 1999) plugin in Geneious Prime. These primers were used in PCR reactions (master mix per reaction: 1U MyFi Taq, 1X final concentration of MiFi PCR Buffer, 1 mM primer concentration; conditions: 95 °C for 5 mins then 30 cycles of 95 °C for 30 s, 60 °C for 30 s, 72 °C for 1 min followed by 72 °C for 5 min) to amplify *Cardinal* homology arms in *An. farauti* equivalent to those used in *An. gambiae* by Carballar-Lejarazú et al. 2020—see Table S1 for details on primers used. The regions amplified span approximately 550 bp upstream of the start codon of the *Cardinal* gene to 1227 bp into the third exon. The expected amplicon sizes of the primers used are approximately 800 bp, 960 bp and 875 bp respectively. One putative 21 bp insertion was found in an *An. farauti* individual sampled from the northern Solomon Archipelago (Choi-4) and no other indels were present in exonic regions sequenced, however indels were common in non-coding and intronic regions.

Based on the population structure observed using microsatellites, we amplified a set of individuals from all populations to assess population level variation in the homology arms of pCO-37. We also isolated the *Cardinal* gene from the *An. farauti, An. irenicus* and *An. hinesorum* individuals for which we generated whole genome sequence data (described above). We achieved this by calling variants on each individual using bcftools (Danecek et al. 2021) mpileup (baseQual = 20, mapQual = 20, snpDepthMinCount = 5) on the genomic region where the *Cardinal* gene is located (supercontig KI915040: 7792713–7795879). This was performed after quality control, mapping to the *An. farauti* genome release 54 (Neafsey et al. 2015) and duplicate removal as described above. *Cardinal* sequences obtained from both methods were aligned using ClustalX (Larkin et al. 2007). Due to a non-overlap between Sanger-sequenced products following quality editing of chromatograms in some individuals between primer sets FAR_CARD_HM2 and FAR_CARD_HM3 (Supplementary Table 2), we removed a small part (22 bp) of the *Cardinal* homology arm sequence from the final alignments. A maximum likelihood phylogeny was built using the IQ tree webserver (Nguyen et al. 2015) which used ModelFinder (Kalyaanamoorthy et al. 2017) to identify the best-fit nucleotide substitution model (TIM2 + F + R4) and ultrafast bootstrapping (Hoang et al. 2018) was performed for 1000 bootstrap replicates. We then assessed the possibility for design of sgRNA sites within the *Cardinal* gene that would be unique to local populations of *An. farauti* based on the alignment and the known population structure of *An. farauti*.

## Results

### Microsatellite population structure, relationships, and diversity

**Fig. 3:**
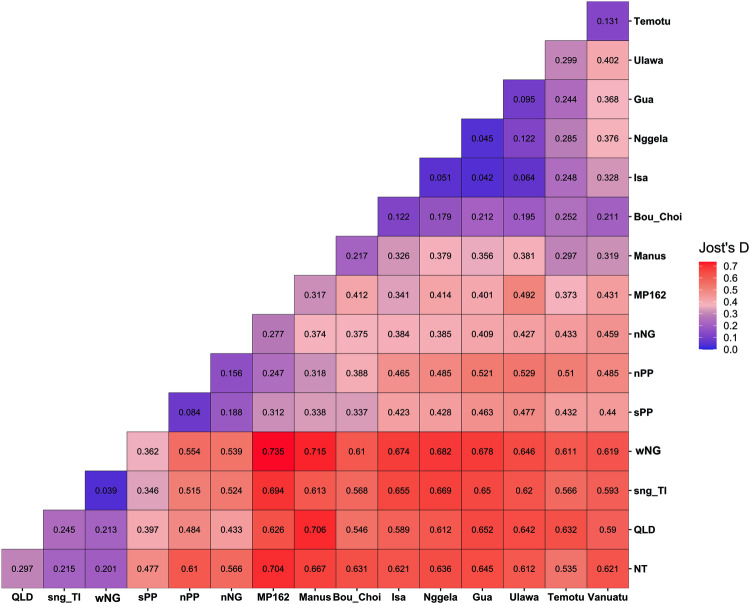
Population relationships based on Jost’s D—pairwise matrix. Higher Jost’s D values indicate greater levels of population differentiation. Populations are abbreviated in the same way above as throughout the manuscript: NT Northern Territory, Australia, Qld Queensland, Australia, TI Torres Strait Islands, sNG southern New Guinea, wNG western New Guinea, sPP southern Papuan Peninsula, nPP northern Papuan Peninsula, nNG northern New Guinea, Manus Manus Island, Bou Bougainville Island, Choi Choiseul Island, Isa Santa Isabel Island, Ng Nggela Island, Gua Guadalcanal Island, Ulawa Ulawa Island, Tem Temotu, Van Vanuatu.

STRUCTURE is a Bayesian clustering program which aims to identify population genetic structure in a data set. It does this by assigning individuals to K groups, each of which are in (or close to) Hardy-Weinberg equilibrium. Initial STRUCTURE analysis performed on the whole dataset identified three distinct genetic groups of *An. farauti* that are found in different geographic regions, and K3 was the most likely value using the Evanno method (Evanno et al. 2005). The Evanno method, although often overly conservative (Janes et al. 2017), seeks to identify the most likely number of groups, K, identified by programs such as STRUCTURE by assessing changes in likelihood (or posterior probability) between increasing levels of K. The three groups identified using the Evanno method are: (Group 1) northern Australia, Torres Strait, western New Guinea (Papua province) and southern New Guinea, (Group 2) the Papuan peninsula and northern New Guinea and (Group 3) Bismarck and Solomon Archipelagos and Vanuatu (Fig. 2A). These results are supported by Jost’s D values estimated between each location sampled when analysing the entire dataset for the 11 loci genotyped (Figs. 2C and 3). Jost’s D is analogous to the more commonly known fixation index, *F*_*ST*_, but ideally suited to highly variable markers such as microsatellites as it is less susceptible to bias from multiallelic effects (Jost 2008). Results from AMOVA show that 21.55% of genetic variance sampled in the microsatellite dataset can be attributed to between region genetic differences between the three major groupings identified in the initial STRUCTURE analysis (Table 2). This is the second highest category of variance assigned by the AMOVA, and only within sample variance (55.61%) explains a greater proportion of genetic variance in the dataset. However, 10.85% of variance is explained by between population within region differences, and only 3.18% is explained by between site within population differences, providing support for the populations assigned by STRUCTURE when running the program on the three regions separately (Fig. 2B).

**Table 2:** Microsatellites—AMOVA.

	Df	Sum Sq	Mean Sq	Sigma	%	Phi
Bt. Region	2	1818.94	909.47	1.88	21.55	0.22
Bt. Pop w/in Region	15	1001.84	77.06	0.95	10.85	0.14
Bt. Site w/in Pop	29	364.53	11.76	0.28	3.18	0.05
Bt. Samp. w/in Site	566	3611.82	6.38	0.77	8.81	0.14
W/in Samp	613	2970.25	4.85	4.85	55.61	0.44
Total	1225	9767.37	7.97	8.71	100	–

Allelic richness was generally higher in populations from Group 2 than in other groups (Table 3), apart from MP162 which constitutes one site on a small island which was considered its own population due to obvious differentiation from populations on mainland New Guinea. This population along with the Manus Island population show some affinity with Group 3 from the Solomon Archipelago (Fig. 2A), and do not clearly fit in to any of the assigned grouping based on Jost’s D (Figs. 2C and 3). Surprisingly, allelic richness values are similar between populations in Group 1 and Group 3. Lower values of allelic richness in populations from Group 1 compared to Group 2, could reflect the less stable and more seasonal climate found in this region, which may force populations through periodic bottlenecks. This is especially pronounced in the Northern Territory population which has the lowest allelic richness of any population in Group 1 (Table 2). Lower values in Group 3 likely reflect smaller populations which have been through historical genetic bottlenecks associated with founder effects. This is most obvious in the Vanuatu and Temotu populations which have the lowest allelic richness of any populations and are the most geographically remote populations in Group 3. Genomic locations of microsatellite loci on the *An. farauti* reference genome can be found in Table S1.

**Table 3:** Microsatellites—allelic diversity statistics.

Region	Population	*N*	Mean allelic richness	Private alleles	N sites
GROUP 1	NT	20	2.342	1	4
	Qld	33	2.486	4	4
	TI, sNG	111	2.527	8	8
	IJ	18	2.981	1	2
	sPP	78	3.881	16	6
GROUP 2	nPP	54	4.1	11	5
	MP162	9	2.498	1	1
	nNG	68	3.886	33	9
GROUP 3	Manus	7	2.498	4	1
	Bou, Choi	23	2.404	0	2
	Isa	20	2.322	0	1
	Ngg	27	2.656	1	1
	Gua	94	2.494	3	1
	Ulawa	10	2.197	0	1
	Temotu	29	1.857	0	1
	Van	12	1.242	2	1

**Fig. 4:**
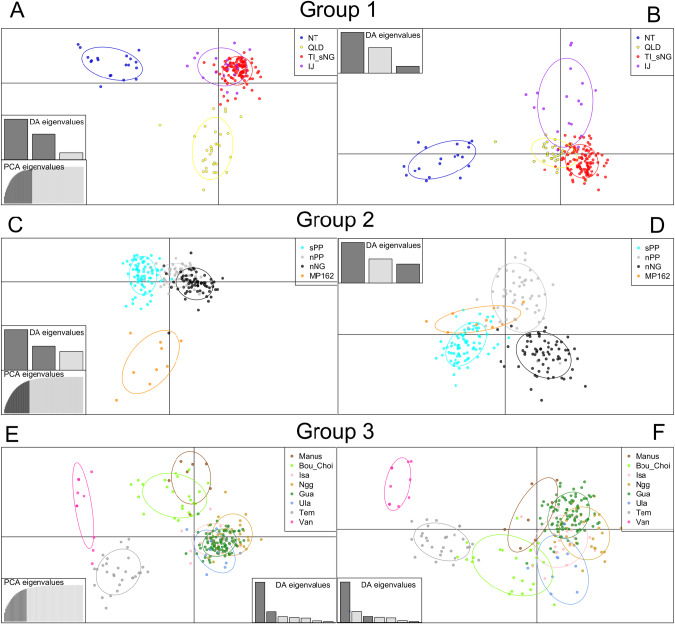
*Anopheles farauti* microsatellite population structure and relationships—DAPC. **A** Shows DAPC scatterplot from group 1, DA1 (x-axis) vs DA2 (y-axis). **B** Shows DAPC scatterplot from group 1, DA1 (x-axis) vs DA3 (y-axis). **C** Shows DAPC scatterplot from Group 2, DA1 (x-axis) vs DA2 (y-axis). **D** Shows DAPC scatterplot from group 2, DA1 (x-axis) vs DA3 (y-axis). **E** Shows DAPC scatterplot from Group 3, DA1 (x-axis) vs DA2 (y-axis). **F** Shows DAPC scatterplot from group 3, DA1 (x-axis) vs DA3 (y-axis).Population abbreviations used as in Table 1 and through the manuscript: NT Northern Territory, Australia, Qld Queensland, Australia, TI Torres Strait Islands, sNG southern New Guinea, wNG western New Guinea, sPP southern Papuan Peninsula, nPP northern Papuan Peninsula, nNG northern New Guinea, Manus Manus Island, Bou Bougainville Island, Choi Choiseul Island, Isa Santa Isabel Island, Ng Nggela Island, Gua Guadalcanal Island, Ulawa Ulawa Island, Tem Temotu, Van Vanuatu.

When running STRUCTURE on Group 1 separately, K2 was the most likely number of groups under the Evanno method. The Evanno method often underestimates K and has been found to be biased towards K2 (Janes et al. 2017). At K2, Australia forms a group distinct from Torres Strait and New Guinea. However, the likelihood method supports additional population structure, with K8 being most likely. We present data for K4 as these results make the most biological sense, and at this K value there appears to be clear and meaningful population structure between groups. At K4, Queensland (Qld, sites 1–8, Fig. 1, Table 1) and Northern Territory (NT, sites 9–17) appear as distinct populations in Australia, while samples from Torres Strait (TI, sites 18–20) and southern New Guinean (sNG, sites 23–28) form another group (Fig. 2B, top panel). The population from western New Guinea (wNG, sites 21–22) appears to be somewhat distinct from southern New Guinea and the Torres Strait, although it is more closely related to this population than to Australian populations, as supported by results at K2 (Supplementary Fig. 1) and pairwise Jost’s D values (Fig. 3). There is some evidence of possible admixture or shared ancestry between the populations from Queensland and southern New Guinea/Torres Strait and between populations in southern New Guinea and Northern Territory. Within the group, values of Jost’s D suggest that Queensland and Northern Territory are the most genetically distinct (Fig. 3) and this is supported by STRUCTURE and DAPC results (Figs. 2B and 4). All populations in this group show evidence of private alleles (one in Northern Territory, four in Queensland, eight in southern New Guinea/Torres Strait, and one in western New Guinea, Table 2).

In the Papuan peninsula and northern New Guinea group we found that the optimal K was 2 based on the Evanno method and K9 for the likelihood method. We present STRUCTURE results for K4 as at this level of K there is the clearest population structure between groups broadly corresponding to geographical areas (Fig. 2B). In this part of New Guinea, it appears that population structure is not as clearly defined as in other regions, at least based on the STRUCTURE analysis for microsatellite markers used in this study. This is somewhat surprising given that populations from this region, as defined by the STRUCTURE analysis at K4, show the highest number of private alleles of any observed in this study. Evidence of admixture is present between the southern (sites 29–34) and northern Papuan Peninsula (sites 35–38, 40) populations, particularly near the population boundaries (Fig. 2B), and these two populations are the most closely related within this group (Figs. 2C, 3 and 4C, D). The northern New Guinean population (sites 39, 41–43, 45–48) is more distinct from these two populations based on Jost’s D (Figs. 2C and 3) and DAPC (Fig. 4C, D), but it does show some evidence of admixture or shared ancestry with the southern Papuan Peninsula population (Fig. 2B). The MP162 population (site 44), sampled from a small island near the northern coast of New Guinea, is clearly the most distinct within the group (Figs. 2B, 3), and as mentioned above, shows some similarity to Group 3 from the Solomon Archipelago. The northern New Guinean population shows the highest number of private alleles (*n* = 33), followed by the southern Papuan Peninsula population (*n* = 16), then the northern Papuan Peninsula population (*n* = 11) and finally the population from site 45 (MP162) with one private allele sampled.

Within the Bismarck, Solomon Archipelago and Vanuatu populations we find the optimal K to be 2 based on the Evanno method and 8 based on the Likelihood method. K2 is likely an under-representation of population structure in this region as is illustrated by clear population structure at K8 (Fig. 2B). Within this group, STRUCTURE analysis suggests that Manus Island (site 49) forms a clear and distinct population, separate from all other populations in the group. This is supported by Jost’s D values (Figs. 2C and 3) and DAPC (Fig. 4E, F). Bougainville (Bou, site 50) and Choiseul (Choi, site 51) appear to constitute a single population which is the most distinct of the Solomon Archipelago populations (Figs. 2B, C and 3). Although STRUCTURE results show that Santa Isabel (Isa, site 52), Nggela (Ngg, site 53), Guadalcanal (Gua, site 54) and Ulawa (Ula, site 55) all represent distinct populations (Fig. 2B), Jost’s D values outline the very close relationships between samples from these islands (Figs. 2C and 3). Temotu (Tem, site 56) and Vanuatu (site 57) populations are clearly distinct from all other populations (Fig. 2B), and although they are separate populations, they are each other’s closest relatives based on pairwise Jost’s D (Figs. 2C and 3). Despite a clear distinction between populations from Group 3, four populations assigned by STRUCTURE at K8 (Bougainville/Choiseul, Santa Isabel, Ulawa and Temotu) do not contain private alleles in the microsatellite dataset. The Manus population contains the highest number of private alleles in the group (*n* = 4), followed by Guadalcanal (*n* = 3), Vanuatu (*n* = 2) and finally one private allele was sampled from the Nggela population.

### Whole mitogenome and *Cardinal* sequence analysis

**Fig. 5:**
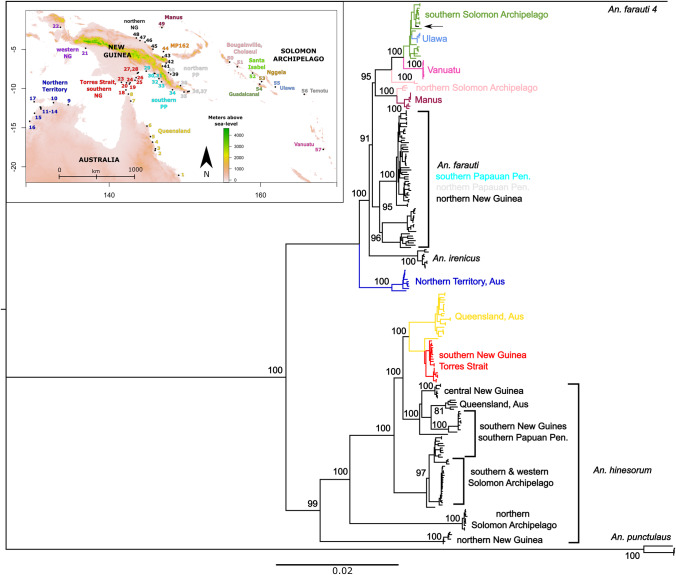
Whole mitogenome phylogeny. The above figure represents a Maximum Likelihood phylogeny which was generated from whole mitochondria (excluding non-genic region) of *An. farauti*, *An. hinesorum* and *An. irenicus*. Node values represent support from 1000 bootstrap replicates. Populations identified using nuclear microsatellites that are monophyletic are shown in colours, as represented throughout the manuscript. *An irenicus* samples mostly form a well-supported monophyletic clade except for the individual highlighted by the arrow.

The assembled mitochondrial genome, excluding the AT rich region was 14,831 bp in length with 22 tRNA genes, an s-rRNA gene, a l-rRNA gene and 13 protein coding genes (37 genes total) and an AT content of 77.5%. Phylogenetic analysis of the whole mitochondrial genome largely agrees with previous studies that have used the mitochondrial COI gene for phylogeography/phylogenetic analyses (Ambrose et al. 2012). As has been shown previously, *An. farauti* is not monophyletic for mitochondrial DNA, with populations from Queensland, Torres Strait and southern New Guinea carrying a mitochondrial genome from *An. hinesorum* that has likely introgressed at some time in the past. Mitochondrial lineage sorting appears almost complete between Queensland and southern New Guinea/Torres strait (Fig. 5), with only one individual from Queensland grouping with the southern New Guinea/Torres Strait clade. Several *An. farauti* populations appear to be monophyletic for mitochondrial DNA including Northern Territory, Manus Island, Ulawa and Vanuatu populations. The Northern Territory population sits basal within *An. farauti*, with *An. irenicus* nested within the species. There is also evidence of recent introgression between *An. farauti* and *An. irenicus* on Guadalcanal where they co-occur, as one *An. irenicus* individual was found carrying an *An. farauti* mitogenome (Fig. 5). One population of *An. hinesorum* from northern New Guinea, which has previously been suggested to be a separate species (Beebe et al. 1996; Ambrose et al. 2012), is supported as being monophyletic and highly differentiated from the rest of the species by the whole mitogenome phylogeny (Fig. 5).

**Fig. 6:**
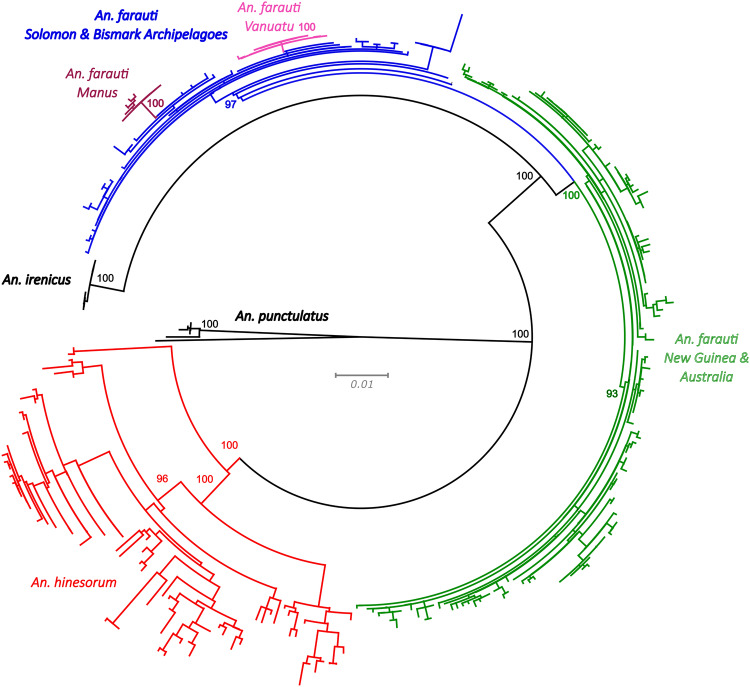
Phylogeny of *Cardina*l gene homology arms. The above figure represents a Maximum Likelihood phylogeny which was generated from sequence data of *An. farauti*, *An. hinesorum* and *An. irenicus* which is homologous to homology arms used in an *An. gambiae* gene drive system. Node values represent support from 1000 bootstrap replicates. Most *An. farauti* samples are coloured by broad geographic region (Solomon Archipelago – blue; Australia and New Guinea – green), as these regions were found to form monophyletic clades. However, individuals sampled from populations in Vanuatu and Manus Islands form monophyletic groups within the Solomon Archipelago clade and are coloured in the same way as they are throughout the manuscript. *Anopheles irenicus* and *An. punctulatus* are both monophyletic and coloured black, while *An. hinesorum* is shown in red.

The phylogeny based on nucleotide sequences of the *Cardinal* homology arms is less resolved than the mitochondrial genome phylogeny, but it did resolve the three sister species, *An. farauti*, *An. hinesorum* and *An. irenicus*, as monophyletic. Within *An. farauti*, there is little phylogenetic signal at this locus, with many populations that are highly differentiated for microsatellites forming unresolved polytomies (Fig. 6). There are two main clades that are resolved within *An. farauti*, one which includes all samples from Manus, the Solomon Archipelago, Temotu and Vanuatu, and another which includes all samples from Australia and New Guinea. There are also two island populations, Manus and Vanuatu, that form monophyletic groups (Fig. 6). The Vanuatu population of *An. farauti* was the only geographically confined group to contain fixed and private SNPs in the *Cardinal* homology arm sequences. In this population, 16 individuals were sequenced, and two SNPs were found that are both fixed and private (sites 731 and 2102, Supplementary Data), with one more that is private and found at very high frequency (one individual sampled was heterozygous—approximately 97% frequency, site 1820). Additional sampling is required to determine the actual frequency of these alleles in the Vanuatu population and could be achieved by sequencing these alleles in hundreds of *An. farauti* samples. The population on Manus Island also contained two SNPs that are fixed, or present at high frequency, and very rare in other *An. farauti* populations. In this, it should be noted that additional sampling is necessary in both the focal and comparator populations to be confident that these alleles are fixed and/or private. Additionally, private alleles were assessed for the entire region sequenced, and due to the limited number of populations containing private alleles we did not assess their suitability as sgRNA targets in a homing-based gene drive.

## Discussion

### Population structure of *Anopheles farauti* in the Southwest Pacific

In this study we build on previous work (Ambrose et al. 2012, 2014) to further characterise the population structure of *An. farauti* through most of its geographic range using nuclear microsatellites and whole mitogenome data. Our analysis of microsatellite data shows that there is strong nuclear population structure between *An. farauti* populations, with genetically distinct populations present in well-defined geographical regions. The strongest level of nuclear population structure separates three distinct groups within the species—one from Australia, Torres Strait Islands, southern and western New Guinea (Group 1), one from the Papuan Peninsula and northern New Guinea (Group 2), and one from the Solomon Archipelago, Manus Island and Vanuatu (Group 3). Within each of these groups, additional substructure was also observed.

Within Group 1, there are two highly distinct Australian populations from Northern Territory and Queensland. Southern New Guinea and Torres Strait Islands (the islands between the Australian mainland and New Guinea) form a third group, with some evidence of admixture between northern Queensland sites and some sites in the Torres Strait, as can be seen in the top panel of Fig. 2B. There is a very close relationship between the populations in western New Guinea and southern New Guinea/Torres strait with possible genetic connectivity between them (Fig. 2). Group 2 contains four populations from the remainder of New Guinea – two populations from the north and south of the Papuan Peninsula (sPP and nPP), northern New Guinea (nNG) and site 44 (MP162). This population sampled from site MP162 is located on a small island approximately 50 km off the coast of northern New Guinea (site 44, Fig. 1) and is the most divergent population within Group 2. There is some evidence of possible gene flow/admixture or recent shared ancestry between other populations in Group 2 (Fig. 2). Further, it should be noted that the southern Papuan Peninsula (sPP) population from Group 2 shares a geographic boundary with the southern New Guinea (sNG) population from Group 1, with no evidence of gene flow/admixture between these populations. Given the genetic distinctness of these populations despite their geographical proximity, they may be locally adapted to climatic or environmental differences between the areas that they inhabit, preventing successful migration between them. In this, we would suggest that the confluence river deltas of the Fly, Wawoi, Kikori and Purari rivers present over 200 km of almost continuous coastal river delta systems and a barrier to this coastally restricted species. Finally, Group 3 consists of island populations of *An. farauti* from Manus Island (PNG) (site 49, Fig. 1), through the Solomon Archipelago to Vanuatu (Table 1, Fig. 1). Microsatellite data suggests that there are eight well defined populations within this groups corresponding to various islands throughout the Southwest Pacific where *An. farauti* occurs. Bougainville and Choiseul islands form a single population, but on all other islands sampled in this study, there are genetically distinct and relatively isolated *An. farauti* populations (Fig. 2). An earlier study through this region comparing microsatellite and mitochondrial COI sequences found some discordance between microsatellites and mitochondrial markers in the location and strength of genetic breaks through these islands suggesting this may be evidence of some male-biased dispersal (Ambrose et al. 2014).

The whole mitogenome phylogeny further expands previous work that used the cytochrome oxidase 1 gene and suggested that an ancient mitochondrial introgression has occurred between *An. hinesorum* (formerly *An. farauti* 2) and *An. farauti* (Ambrose et al. 2012). As can be seen in Fig. 5, all *An. farauti* samples sequenced from populations in Queensland, Torres Strait and southern New Guinea carry a mitogenome that is of *An. hinesorum* descent – originating from this ancient mitochondrial introgression. This evolutionary outcome suggests that there may have been selection pressure for the retention and spread of the *An. hinesorum* mitochondria during a range expansion of *An. farauti*, resulting in complete mitochondrial replacement throughout this part of *An. farauti*’s range. Although mitochondrial introgression appears to be common in other animals, this phenomenon of organellar introgression and replacement through whole geographically defined populations has rarely been observed. One such event of chloroplast replacement in an algal species has been published and this event was associated with the species’ range expansion and was possibly driven by a selective sweep (Neiva et al. 2010). In this, *An. hinesorum* shows highland affinities in PNG (in places occurring at altitudes >1500 m) and its closest cryptic relatives *An. farauti* 5 and *An. oreios* (formerly *An. farauti* 6) occur only in the PNG highlands (Beebe et al. 2015; Bangs et al. 2015). Thus, some populations of *An. hinesorum* may have evolved a mitochondrial phenotype which provides a selective advantage in cooler climates, and this hypothesis warrants further exploration. Interestingly, samples from Northern Territory sites are not a part of this southern introgressed lineage and form a monophyletic group which sits basal within *An. farauti* in the mitogenome phylogeny. They are also more distantly related to other *An. farauti* populations than is *An. irenicus*. This genetic distinctness, also observed in the nuclear (microsatellite) loci, presents an hypothesis that *An. farauti* in the Northern Territory may be a separate cryptic species in the *Anopheles punctulatus* group (which currently has 13 recognised species) (Beebe et al. 2015). The genetic distinctness between sites from Northern Territory and Queensland also further supports the biogeographic significance of the Carpentarian Gap, which has been shown to influence the distributions of numerous other species in northern Australia (Bowman et al. 2010; Catullo et al. 2014; Pepper et al. 2017). Populations of *An. farauti* from Manus Island, Ulawa Island and Vanuatu appear to have had sufficient time in isolation for complete mitochondrial lineage sorting to occur although larger sample sizes are required to be confident of this finding. There is also evidence of a possible recent mitochondrial introgression between *An. farauti* and *An. irenicus* on Guadalcanal in the Solomon Archipelago as one *An. irenicus* sample appears very closely related to *An. farauti* from Guadalcanal.

### *Anopheles farauti* population structure and distribution provides a system for evaluating gene-drives in the field

*Anopheles farauti* is a primary malaria vector in the Southwest Pacific (Cooper and Frances 2002; Cooper et al. 2009) with some island populations of the species showing behavioural adaptation to traditional vector interventions (insecticide-treated nets and indoor insecticide spraying) (Bugoro et al. 2011, 2014; Russell et al. 2016). This nature of this adaptation involves a shift in feeding behaviour from all night indoor biting to early evening outdoor biting, meaning that the species can now avoid indoor insecticide applications (Bugoro et al. 2011, 2014). Thus, alternative approaches to control malaria are necessary in this region. Recent advances in the development of gene-drive systems make them a compelling prospect for modifying mosquito populations for disease mitigation or elimination (Pickar-Oliver and Gersbach 2019; Rode et al. 2019; Bier 2022).

The precision afforded by CRISPR is enabling researchers to make more specific and predictable changes to the genomes of organisms, allowing for additional flexibility and a broader range of applications when designing gene-drives (Champer et al. 2016; Nash et al. 2019; Oberhofer et al. 2020). These engineered organisms could be some of the most powerful and effective tools for combatting pests and diseases, with applications in both population eradication (for example of agricultural pests or vectors of disease) (Hammond et al. 2016, 2021; Kyrou et al. 2018; Simoni et al. 2020), and population modification (for example, systems can be engineered to modify a disease vector population so that that cannot transmit pathogens causing disease) (Gantz et al. 2015; Pham et al. 2019; Noble et al. 2019; Adolfi et al. 2020; Carballar-Lejarazú et al. 2020). However, despite the promise of gene-drives for controlling pests and diseases, CRISPR-based homing gene-drive systems have not yet been released in a field setting. This is partly due to a lack of understanding about how they will spread and impact natural populations and ecosystems. Additionally, the likelihood of gene-drives spreading across geo-political borders makes regulation and governance difficult. To address this gap in knowledge, research groups are modelling how gene-drives are likely to spread once released. The caveat of many of these models is that they rely on assumed, user-defined parameters which are informed by laboratory-based small cage trials and theoretical population dynamics (Eckhoff et al. 2017; Noble et al. 2017; North et al. 2020; Sánchez et al. 2020; Wu et al. 2021; Beaghton and Burt 2022; Leung et al. 2022; Champer et al. 2022). Recent large cage trials have allowed for the assessment of gene-drive performance in a setting closer to field conditions (Hammond et al. 2021), but these cage scenarios are an incomplete representation of how a gene-drive may spread in a full field setting. This is especially the case due to gaps in knowledge around male and female mosquito dispersal, host seeking, mate seeking and other aspects of each mosquito species’ lifecycle.

There are several promising prospects for limiting the spread of engineered gene-drive organisms, including the use of double drives which would target two separate loci (one conserved and one private to target populations). This may provide an effective way to confine gene-drives to local populations (Willis and Burt 2021), potentially providing a safer route to initiate field trials. Since *Cas* enzymes are tolerant to polymorphisms in the sgRNA recognition sequence, targeting private alleles present in the PAM sequence itself may be the most reliable way to mitigate this risk (Rabinowitz and Offen 2021). There is however a trade-off between targeting (functionally) conserved sites to minimize resistant alleles evolving or being tolerated and targeting variable sites that may be different between populations. In this regard, it may be possible to find sites that are locally selected for (and functionally conserved) in specific populations but not in others. Other prospects for limiting gene-drive spread include self-limiting drives such as daisy drives (Noble et al. 2019), chemically controllable gene-drives (Chae et al. 2020) and genetically controlled anti-CRISPR molecules (Taxiarchi et al. 2021). To test these approaches, it makes sense to perform small scale field releases in systems with strong population structure, present in genetic groups occupying small geographic regions, rather than trialing gene-drive releases in more panmictic species. As shown in this paper, *An. farauti* exhibits very strong genetic and geographic structure, making it a good system for field trialing gene-drive strategies, which target specific and confined populations. Further, the species has an essentially linear (coastal) distribution which provides practical advantages for both modelling and monitoring releases. Our current population genetic understanding of *An. farauti* also provides valuable information for directed field sampling when performing whole genome sequencing to explore potential guide RNA target discovery for future gene-drives that could be constrained to specific, geographically restricted populations by targeting private alleles.

As well as characterising the population structure of *An. farauti* using microsatellite and mitochondrial data, we assess sequence variation in the 2.2 kb *Cardinal* gene homology arms used in the previously developed pCO-37 *An. gambiae* gene-drive system (Carballar-Lejarazú et al. 2020). This was done to assess the suitability of the *Cardinal* locus and a modified version of the pCO-37 plasmid (Carballar-Lejarazú et al. 2020) for developing a strain of *An. farauti* for localized population modification. A recent study targeting the *Cardinal* gene in *An. gambiae* and *An. coluzzi* successfully integrated and expressed anti-malarial effector molecules targeting the *Plasmodium falciparum* parasite ookinetes and sporozoites. Based on the results of these laboratory-based experiments, models suggested that using gene drive strains similar to these for population replacement could be effective in reducing human malaria by more than 90% three months following release (Carballar-Lejarazú et al. 2023). For a drive aimed at population replacement to be designed around this locus to be specific to confined populations, high frequency (ideally fixed) private alleles would need to be present in *An. farauti* target populations. It should be noted that for a population suppression drive, any alleles which are resistant to the drive would be problematic meaning that identifying fixed and private alleles would be essential.

High frequency private alleles for the *Cardinal* gene were only found in the most remote and southern Pacific Island population from Vanuatu. Thus, for most populations, a drive designed around the *Cardinal* gene in *An. farauti* is likely to function as a global-drive system, as it would in *An. gambiae* (Carballar-Lejarazu et al. 2020). However, it may be possible to use a modified *Cardinal* plasmid in conjunction with an additional *Cas9*-sgRNA complex inserted elsewhere in the genome, at a site that is fixed and private in specific populations and designed to cut the conserved *Cardinal* locus (i.e., a double drive – as described in Willis and Burt 2021). To facilitate the identification of suitable loci (fixed and private alleles) additional population genomic data for *An. farauti* is recommended using the microsatellite data as a guide for further field sampling. Subsequently this data could be used to identify other loci in the genome around which to design a gene-drive system for specific modification of genetically isolated and regionally restricted *An. farauti* populations. When identifying these loci, it will also be vital to assess interspecific variation at sgRNA target loci to avoid any potential for gene-drive escape into closely related *Anopheles* species, for example see (Connolly et al. 2022).

### Conclusions, limitations and future directions

This study aimed further investigate the population genetics of *An. farauti* and assess the feasibility of adapting an existing gene drive target from *An. gambiae* (targeting the *Cardinal* gene) to a confinable gene drive in *An. farauti*. To achieve this, we sequenced the *Cardinal* gene from numerous *An. farauti* individuals, and sister species, from genetically and geographically distinct populations. We then searched for fixed and private alleles at the *Cardinal* locus which could be used as population specific gene drive targets. Only one population was found to contain fixed alleles in the data presented, although the sample size per population was relatively low in this study. Additional sampling is required to be more confident that the alleles identified are indeed private to the population.

The extensive population genetic structure observed in this study suggests that *An. farauti* provides a counterpoint to original gene drive exploration with *An. gambiae* and *An. coluzzi* in Africa as well as a new system for engineering gene drives that could be confined to local populations by using sgRNA targets unique to specific populations (private alleles). The species’ coastal distribution also makes it an attractive prospect in this regard as the spread of drive alleles could be modelled in a what is essentially a single dimension prior to release. Because of this strictly coastal distribution, monitoring drive spread would also be simpler following a release. Additionally, the presence of genetically isolated populations in Australia would allow the development of a gene drive to target populations for field trials in a developed country with well-established and robust regulatory processes. Although there would be no epidemiological outcome from such trails (as there is no malaria endemic in Australia), they would enable the initial assessment of gene drive behaviour in a natural field setting within a developed country. As well as this, releasing in a first world and geographically isolated country such as Australia, permits the identification of any potential risks and may facilitate better building of public trust in the technology prior to it being taken to developing countries. A similar release model was implemented and shown to be effective with the initial release and subsequent population replacement of *Wolbachia* infected *Aedes aegypti* in north Queensland (Hoffmann et al. 2011; Ogunlade et al. 2023). These populations have persisted to this day and remain effective in mitigating Dengue risk in the region (Ross et al. 2022; Ogunlade et al. 2023).

### Supplementary information

Supplementary Fig 1, and Tables 1 and 2

## Data Availability

Data used in this study are available from **UQ eSpace**: 10.48610/7d691e8.
