# Supplementary material for: Genetic and geographic population structure in the malaria vector, Anopheles farauti, provides a candidate system for pioneering confinable gene-drive releases
Source: Heredity (Edinb). 2024 Mar 18;132(5):232–46. doi: 10.1038/s41437-024-00677-2 (PMC11074138; doi:10.1038/s41437-024-00677-2)
Supplement: Supplementary file 1 — Supplementary Fig 1, and Tables 1 and 2 [file 41437_2024_677_MOESM1_ESM.pdf]

Supplementary Material

Supplementary Figure Legend

Supplementary Figure 1: ***Anopheles farauti*** **microsatellite population structure and relationships – Bayesian clustering (STRUCTURE).** The top panel shows STRUCTURE results for Group 1 at K=2 and the bottom panel shows STRUCTURE results for Group 2 at K=2. Sites and regions from Table and Figure 1 are shown below each plot.

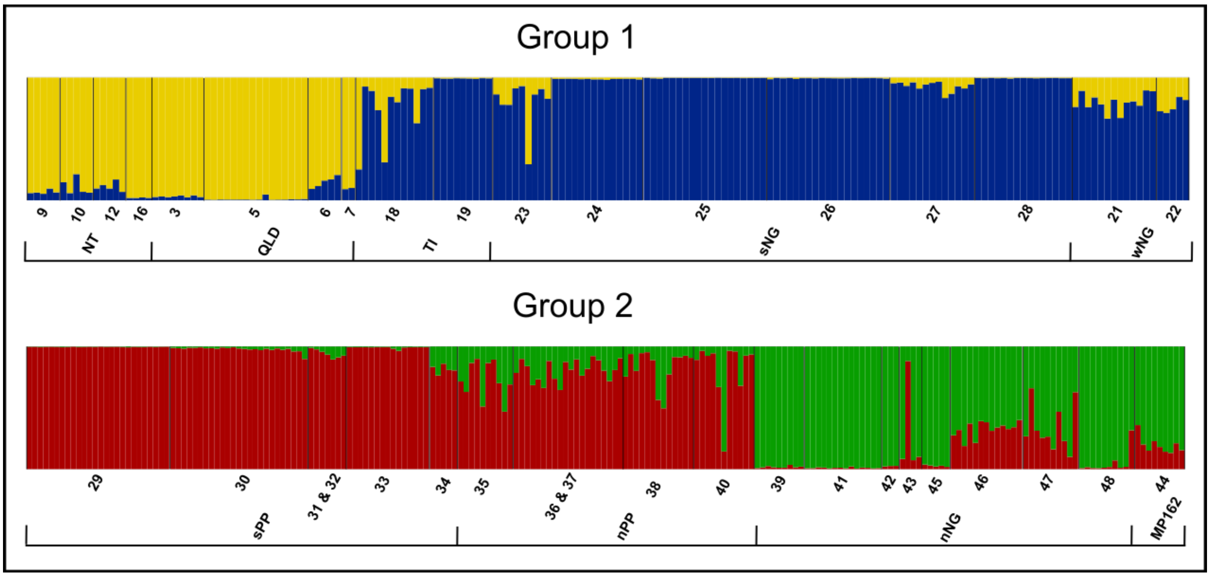

Supplementary Tables

Supplementary Table 1: Microsatellite primers and genomic location

PRIMER NAME	FORWARD SEQUENCE	REVERSE SEQUENCE	GENOMIC LOCATION
**FARDI-5**	GTTGGTGCGATGGTGTAAGG	TGGTCTGAAACTGTTGCGTG	KI915044 (4,381 Mbp)
**FARDI-11**	ATGCTCTTTCGGTGTTTGCG	GCGTTTGAAATCGCTGCTTC	KI915043 (12,205 Mbp)
**FARDI-12**	GGGTGCTAAATGTCTCAGCG	CATGGGCTGCAGTATGTTCG	KI915040 (12,528 Mbp)
**FARDI-14**	GTGAGCGCGTGATATTCCAC	ATCGGTCAGTAGGGTTAGCG	KI915058 (0,462 Mbp)
**FARTRI-1**	ATGTGGTCGGCTGTTTATGG	GCGTAAACACATGTGCGATC	KI915040 (12,647 Mbp)
**FARTRI-5**	TTTAGCTGCAACACGACGAC	CGCGAGAAAGAGGAAACCAC	?
**FARTRI-8**	CAGTTCATCATTCCCACGGC	GTGGATTCCGAAGAGCAACG	KI915048 (5,124 Mbp)
**FARTRI-14**	TTGTGCGGGATGAAATAGCG	AGGCAAAGAGTCGGTCAGAG	KI915048 (1,289 Mbp)
**FARTRI-15**	GGGCATGTGTTTCGCTAGTG	GTTCGCACTCCACGGTTTAG	KI915044 (5,189 Mbp)
**FARTRI-19**	AGATTAAATGTGGATTCAGAAG	CTTCACATAACGCCAGATCG	KI915065 (0,365 Mbp)
**FARTRI-24**	TCTAGGGCGTGCAGTGTG	CAAAGGTGGCGCAATGGG	KI915041 (15,920 Mbp)
**FARTRI-29**	GCAGACGGTCTTCATTGAGC	ACGTTGGCTAGAATTGCGAG	KI915045 (5,394 Mbp)

Supplementary Table 2: *Cardinal* homology arm primers

PRIMER SET	FORWARD	FORWARD SEQUENCE	REVERSE	REVERSE SEQUENCE
**FARCARD_HM1**	**Card_525F**	TGACGGTCAATGAGAACACTTTTCGG	**Card_1325R**	TGCGGAACTCACATGATGGAGGC
**FARCARD_HM2**	**Card_1176F**	GTTGACGTCCGGTGATGTTTCCG	**Card_2134R**	GATCACTGTGGTAGGACGGGCG
**FARCARD_HM3**	**Card_1980F**	CGAGTACGGTGTCGCGATGATCC	**Card_2855R**	GCGTTGTACGGGTCGTCGTGAG
